# Optimization of Diffusion MRI With Consideration of the Signal Decay in Biological Tissues

**DOI:** 10.1002/mrm.70346

**Published:** 2026-03-19

**Authors:** Stefan Kuczera, Stephan E. Maier

**Affiliations:** ^1^ Department of Radiology, Institute of Clinical Sciences Sahlgrenska Academy, University of Gothenburg Gothenburg Sweden; ^2^ Department of Radiology, Brigham Women's Hospital Harvard Medical School Boston Massachusetts USA

**Keywords:** diffusion MRI, noise, optimization, prostate

## Abstract

**Purpose:**

Devising methodology to characterize and optimize acquisition schemes for biological tissues, such as the prostate, that produce model‐based synthetic diffusion‐weighted images and derived model parameters with predictable SNR improvement.

**Methods:**

The averaging effect (AE) in synthetic diffusion‐weighted images obtained through fitting of various signal decay models to signals measured over 21 linearly spaced b‐values between 0 and $2000s/{\text{mm}}^{2}$ is determined with analytic expressions. Similarly, the standard deviation of a retrospective 2‐point ADC fit based on the synthetic data is analyzed. Furthermore, acquisition schemes that achieve constant SNR or constant AE for synthetic images over the same b‐value range are devised by means of numerical optimization using either a custom iterative method or a standard function optimizer. These acquisition schemes are verified by measurements on a phantom with a non‐monoexponential diffusion signal decay combined with a bootstrapping approach to increase the number of data samples.

**Results:**

The dependence of AE on model function and parameters is complex. Repeated measurements at specific b‐values can boost AE locally, while improvements in ADC uncertainty are particularly pronounced for repetitions of the higher b‐value. Optimization of acquisition schemes generally results in discrete b‐values, whereby the number of b‐values corresponds to the number of model parameters. Results from phantom measurements are in agreement with the theoretical predictions.

**Conclusion:**

The presented analytical calculations and numerical optimizations can be useful to improve acquisition schemes under various experimental conditions and clinical needs.

## 1 Introduction

Numerical simulations and calculations are a commonly employed strategy for sequence optimizations in diffusion weighted imaging [1]. In our previous work [2], we presented a novel framework for diffusion‐weighted image and ADC generation in prostate imaging: A large number and wide range of b‐values are acquired without repetitions instead of a small number with multiple repetitions. To account for the lower SNR of the individual b‐value images, function fitting as a model‐driven denoising strategy is applied. From these fits, synthetic images can be reconstructed that are of similar quality to the ones obtained from repeated acquisition with averaging. In the current work, we investigate with simulations how the SNR of these synthetic images depends on the underlying parameters of commonly applied diffusion signal decay models. Calculations are based on the work by Richter [3]. In particular, we show how underlying model parameters influence the SNR of the synthetic images and what effect repeated measurements have on reproducible ADC calculation [2]. Moreover, we investigate how, for specific tissue diffusion signal decays, the acquisition scheme can be modified to generate synthetic images with predictable SNR vs. b dependence, for example, a constant SNR or constant averaging effect (AE) [2] over a certain b‐value range.

## 2 Methods

### 2.1 Modeling Functions

In this section, the model functions studied in this work are introduced. In all cases, $S_{0}$ is the signal intensity without diffusion weighting, that is, at b=0. The monoexponential model function is given by:

(1) $$S(b)=S_{0}e^{-bD_{m}},$$

with $D_{m}$ being the single diffusion coefficient. For the biexponential model, the signal S(b) is described by a biexponential function:

(2) $$S(b)=S_{0}\left(fe^{-bD_{f}}+(1-f)e^{-bD_{s}}\right),$$

where $D_{f}$ and $D_{s}$ represent compartments with fast and slow diffusion with relative signal contribution at b=0 of f and 1−f, respectively. The triexponential function is adding a very fast signal decay often related to blood flow, referred to as intravoxel incoherent motion (IVIM) [4], to the biexponential model:

(3) $$\begin{array}{cc} S(b) & =S_{0}(f_{p}e^{-b(D+D^{*})}+(1-f_{p})\left(fe^{-bD_{f}}+(1-f)e^{-bD_{s}}\right)), \end{array}$$

where $f_{p}$ is the relative signal fraction of the perfusion pool with an aggregate diffusion coefficient $D+D^{*}$. In the case of the kurtosis model, the signal is approximated with a Taylor expansion of the decaying exponential, whereby the series is truncated to a second‐order polynomial in b according to the following equation:

(4) $$S(b)=S_{0}e^{-b{\text{ADC}}_{K}+\frac{1}{6}b^{2}{\text{ADC}}_{K}^{2}K},$$

where ${\text{ADC}}_{K}$ represents the kurtosis apparent diffusion coefficient and K the excess kurtosis factor. The signal decay for the gamma distribution model is given by

(5) $$S(b)=S_{0}\frac{1}{{\left(1+\vartheta b\right)}^{k}},$$

where ϑ and k describe the scale and shape parameter of the associated gamma distribution of the diffusion coefficients, respectively. The stretched exponential results in a signal decay

(6) $$S(b)=S_{0}e^{-{\left(b\text{DDC}\right)}^{\alpha }},$$

where DDC is the distributed diffusion coefficient and α is a stretching coefficient that introduces the deviation from a monoexponential decay.

### 2.2 Analytic Prostate Tissue Models

A biexponential model with two tissue types was considered, that is, one labeled “normal” for normal prostate tissue and one labeled “cancer” for cancerous prostate tissue. Model parameters were selected in approximate accordance with literature reference values without the influence of the IVIM component [5]. For both tissue types the fast diffusion parameter $D_{1}$ was set to the same value of $2.2\;\mu m^{2}/\text{ms}$. Simulation values for the slow diffusion parameter $D_{2}$ and fast signal fraction f for normal and cancer were different, that is, $D_{2}=0.4\;\mu m^{2}/\text{ms}$ and f=0.8 for normal tissue and $D_{2}=0.2\;\mu m^{2}/\text{ms}$ and f=0.6 for cancerous tissue. In the case of kurtosis, gamma distribution and stretched exponential function true model parameters were determined by means of direct fitting to this biexponential tissue model data using 1000 linearly spaced b‐values in the range [0, $b_{\max}$] with $b_{\max}=2000\;s/{\text{mm}}^{2}$ (see Figure S2). For the monoexponential model, $D_{m}$ was calculated from the signal values at 0 and $b_{\max}$ of the biexponential model. For the triexponential model, the IVIM contribution was added to the biexponential model. True model parameters in the case of the experimental phantom data are obtained by model fitting of the experimental data as described in Section 2.6. All models and respective parameters are listed in Table S1.

### 2.3 Analytic Expressions and Optimization

As introduced in Kuczera et al. [2], the averaging effect AE as a function of the b‐value b is defined as

(7) $$\text{AE}(b)={\left(\frac{\sigma_{g}}{\sigma_{y}(b)}\right)}^{2},$$

where $\sigma_{y}(b)$ is the uncertainty at b of a model function S(b) after fitting. It can be calculated using an analytical expression given by Richter [3] (see Appendix Equation (A1)). The standard deviation $\sigma_{g}$ of the underlying Gaussian‐distributed signal noise equals 1 for all simulations. SNR at b=0 in the simulations was set to 20, that is, $S_{0}=20$. The averaging effect is a measure of how many repeated acquisitions would be necessary at a certain b‐value to achieve the same reduction in noise standard deviation as attained by fitting with the model function S(b). As measurement points, 21 linearly spaced b‐values in the range $\left[0,b_{\max}\right]$ with $b_{\max}=2000\;s/{\text{mm}}^{2}$ were used without averaging. The b‐value specific signal‐to‐noise ratio ${\text{SNR}}_{\text{fit}}$ resulting with model fitting is given by

(8) $${\text{SNR}}_{\text{fit}}(b)=\frac{S(b)}{\sigma_{y}(b)}.$$

This means that an increase in AE(b) by a factor x corresponds to an increase in ${\text{SNR}}_{\text{fit}}$ by a factor $\sqrt{x}$.

The standard deviation of the two‐b ADC ($\sigma_{\text{ADC}}$) derived with the retrospective fitting procedure [2] was determined from the parameter covariance matrix C described in Equation (A2) in the appendix. Specifically, the uncertainties in Equation (A3) were set to $\sigma_{y}(b)$ resulting from the model fit in the first step. The SNR of the ADC is then defined as ${\text{SNR}}_{\text{ADC}}=\text{ADC}/\sigma_{\text{ADC}}$. The two‐b ADC was calculated as $\text{ADC}=-\ln\left(S(b_{2})/S(b_{1})\right)/(b_{2}-b_{1})$ with $b_{1}=100\;s/{\text{mm}}^{2}$ and $b_{2}=1000\;s/{\text{mm}}^{2}$.

To generate a constant averaging effect f(b)=AE(b) or constant signal‐to‐noise ratio $f(b)={\text{SNR}}_{\text{fit}}(b)$ with model fitting over a b‐value range $\left[0,b_{\max}\right]$ the following iterative procedure is applied: As an initialization step i=0, a vector $b=(b_{1},\ldots ,b_{N})$ with N=500 linearly spaced b‐values in the range $\left[0,b_{\max}\right]$ is generated and each b‐value is given an equal weight $w^{\left(0\right)}(b)=1/N$, so that the sum of all weights equals 1. Each weight w(b) quantifies the relative amount of measurements at the corresponding b‐value. The b‐value specific standard deviation is consequently $\sigma^{\left(0\right)}(b)=1/\sqrt{w^{\left(0\right)}(b)}$. N is not altered during the optimization. In each step i>0 the weights $w^{\left(i\right)}(b)$ are updated and normalized:

(9) $$w^{\left(i\right)}(b)=\text{norm}\left(w^{\left(i-1\right)}(b)/f(b,\sigma^{\left(i-1\right)})\right),$$

where $\text{norm}(v)=v/\sum_{j}v_{j}$. The standard deviation is then given by:

(10) $$\sigma^{\left(i\right)}(b)=1/\sqrt{w^{\left(i\right)}(b)}.$$

This procedure is used to minimize the cost function

(11) $$\text{cost}(x)=\frac{1}{N}\underset{j}{\sum }{\left(x_{j}-\frac{\sum_{k}x_{k}}{N}\right)}^{2},$$

with $x=f(b,\sigma^{\left(i\right)})$. Iteration is stopped when the relative change in $d^{\left(i\right)}=\text{cost}(f(b,\sigma^{\left(i\right)}))$ is small:

(12) $$\frac{d^{\left(i\right)}-d^{\left(i-1\right)}}{d^{\left(i-1\right)}}<10^{-6}.$$

This method shall be referred to as the “inverse method”. As an alternative approach, the cost function cost(f(b,σ(b))) is minimized using a numerical optimizer implemented in SciPy using the “trust‐conv” algorithm. This approach shall be referred to as the “SciPy method”.

In the resulting $w^{\left(\text{final}\right)}(b)$ peak positions $b_{\text{peak}}(i)$ are identified and the peak integral is computed by summation over an appropriate range of b‐values centered around $b_{\text{peak}}(i)$. The width of the b‐value range was $20\;s/{\text{mm}}^{2}$ to $50\;s/{\text{mm}}^{2}$ depending on the width of the local peak and the distance to adjacent peaks. To discretize the b‐value distribution, the number of measurements at b=0 is either set to 1, 5, or 10, depending on the given b‐value distribution. For the other peaks, the number of measurements is given by the rounded value of the peak integral ratio with respect to b=0 times the number of measurements at b=0.

### 2.4 Non‐Monoexponential Phantom

The phantom used for experimental verification consisted of two vesicle‐in‐water emulsions composed of cetearyl alcohol (CA), behentrimethyl ammonium chloride (BTAC), and stearylamidopropyl dimethylamine (SA) at molar ratios of 7:1:1 with w/w ratios of 0.5% (CSB05) and 1.0% (CSB10) [6]. The samples exhibit non‐monoexponential MR diffusion signal decay characteristics similar to prostate tissue [7] with CSB05 imitating normal prostate tissue and CSB10 imitating cancerous prostate tissue. Details regarding the sample container can be found in the supportive information, along with a 2D MR image with highlighted ROIs (Figure S4).

### 2.5 MRI Scans

Phantom measurements were performed on a 3T MRI scanner (Premier; GE Healthcare, Milwaukee WI; software release MR30.0) equipped with a flexible 21 channel array RF coil and a magnetic field gradient coil with a nominal maximum gradient strength of 80mT/m and a maximum slew rate of 200mT/m/ms. Two modifications were added to the vendor‐provided diffusion sequence. First, the maximum number of acquisitions for the applied DTI mode, which reads b‐values and diffusion encoding directions from an input file, was increased to 1000. Second, the requirement of either using dual echo or field monitoring was removed to turn both options off during scanning. Field monitoring was tested, but created noticeable signal oscillations over time. Diffusion encoding direction was set to the slice direction for all b‐values. Only a single 5mm coronal slice was acquired to avoid any cross‐talk between slices. Bottle axes were aligned with the slice encoding direction, and the slice position was set close to the center of the bottles. Sample temperature was around $22^{\circ }C$ and monitored both before and after the measurement. Sample heating during the experiment was not expected, as only one excitation per TR was performed. Three scan series with the following temporal acquisition order were performed (b‐value and number of acquisitions given); Series 1: (0, 250), (10, 250), (100, 250), (2000, 250), Series 2: (0, 30), (1000, 240), (2000, 700), (0, 30), and Series 3: (0, 30), (400, 100), (500, 100), (1200, 250), (1300, 250), (2000, 240), (0, 30). Choice of b‐values was in accordance with expected b‐value results for optimized acquisition schemes. The duration of each series was approximately 33min. Between scans, a prescan was performed manually to ensure that acquisition parameters such as receiver gain stayed constant. Other scan parameters were as follows: TR, 2000ms; TE, 63.8ms; FOV, 340mm×340mm; encoding matrix size, 110 (frequency) × 110 (phase); image matrix size, 256 (frequency)×256 (phase); parallel coil acceleration (SENSE), 2.

### 2.6 Postprocessing MRI Data and Bootstrapping

The first two images of each subseries at a certain b‐value were excluded from further analysis. Moreover, although for b=0 and $2000\;s/{\text{mm}}^{2}$ multiple subseries were measured, only the subseries with the largest amount of measurements were included in the analysis, as this simplified drift correction. Thus, the additional data from the other series were regarded as redundant (see Figure S3). For both bottles, a circular ROI, excluding pixel and bottle edges, was defined (Figure S4). For each b‐value, a second‐order polynomial was fitted to the series of mean ROI signals obtained with the repeated acquisitions. These polynomials were then used for pixel‐wise signal drift correction for each b‐value and ROI.

Experimental data is used to verify the optimized acquisition schemes for constant ${\text{SNR}}_{\text{fit}}$ for three model functions. However, true model parameters need to be adapted for the experimental case. For the monoexponential and kurtosis model function, true experimental model parameters could be computed by fitting the ROI averaged values at the respective analytically optimized b‐values, as these were independent of the actual true model parameter. Particularly, optimized b‐values were the same for the normal and cancer tissue models. On the other hand, for the biexponential function, this was not the case. Therefore, the ROI‐averaged signal was first fitted with all measured b‐values to get an initial estimate of the true model parameters and to compute a set of optimized b‐values. Starting with these optimized b‐values, model parameters and optimized b‐values were calculated multiple times in an iterative fashion until a steady state was reached. Experimental model parameters are listed in Table S1. From the drift‐corrected signal, 1000 signal realizations were created for each pixel by means of bootstrapping and signal averaging, according to the respective optimized experimental acquisition scheme. As the experimental data were generally not acquired at exactly the optimized, analytically derived b‐values, the closest measured b‐values were used instead without altering the number of averages. Example code is available at https://github.com/dMRI‐GU/Optimize‐dMRI‐AE‐SNR.

## 3 Results

**FIGURE 1:**
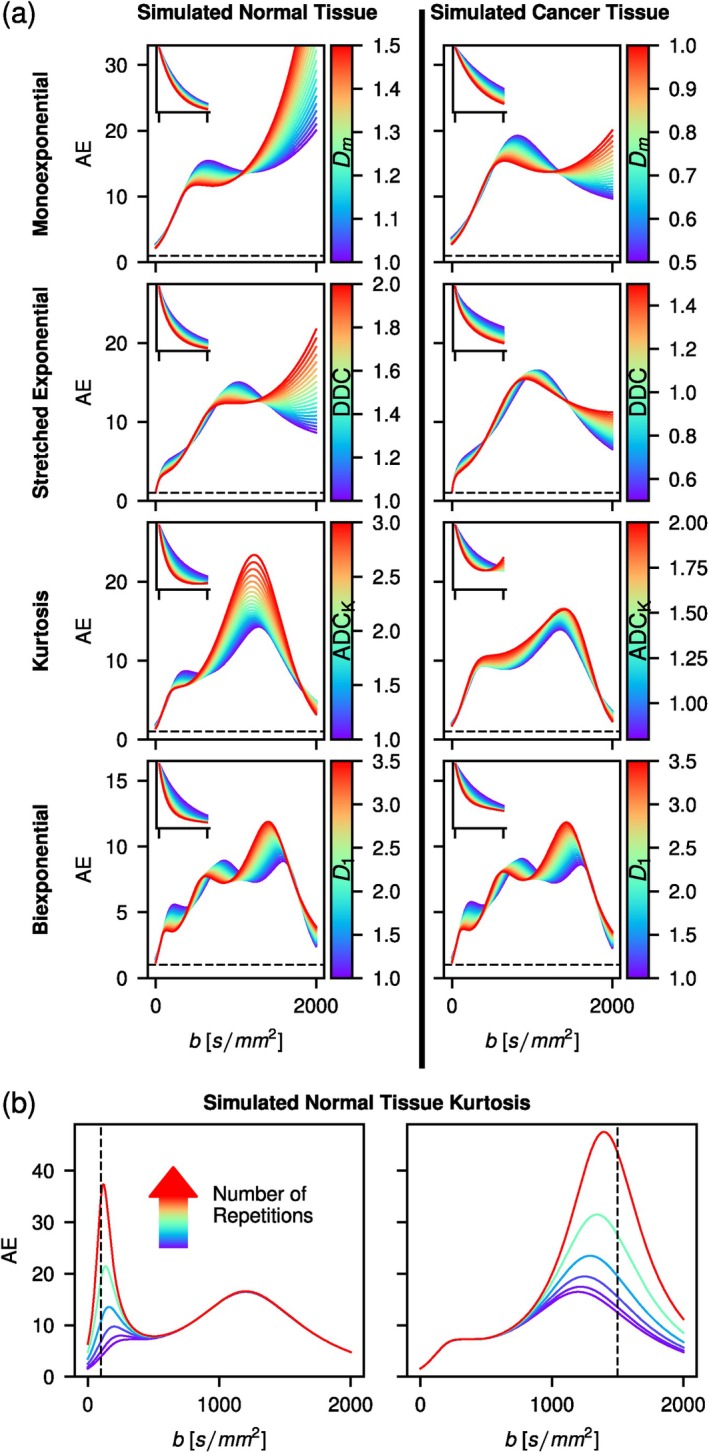
(a) Averaging effect (AE) that results in the two simulated tissue types for various model functions with a single model parameter varied in each plot. The varied model parameter is indicated in the colorbar. The inset shows corresponding signal decay curves for the same b‐range as the main plots. Model parameters are found in Table S1. Dashed lines indicate AE = 1. Note that an increase of AE(b) by a factor x corresponds to an increase in ${\text{SNR}}_{\text{fit}}$ by a factor $\sqrt{x}$. (b) AE for the kurtosis function with simulated normal tissue type at repetitions of 1, 2, 4, 8, 16, and 32, selectively performed at b‐values of 100 (left) and $1500\;s/{\text{mm}}^{2}$ (right). Dashed lines indicate repeated b‐value.

The averaging effect AE for the studied models as a function of b‐value and model parameters is shown in Figure 1a. Generally, the averaging effect is higher towards the center of the b‐value range, with some exceptions for the monoexponential and stretched exponential functions. Furthermore, as also evident in Figure S1, the number of local maxima equals the number of model parameters, not counting $S_{0}$. In Figure 1b, the averaging effect is shown for an increased number of measurements at a b‐value of 100 or $1500\;s/{\text{mm}}^{2}$ for the kurtosis model in the case of simulated normal tissue. Averaging at a certain b‐value increases the AE function locally, but does not have an effect over the whole range of b‐values.

**FIGURE 2:**
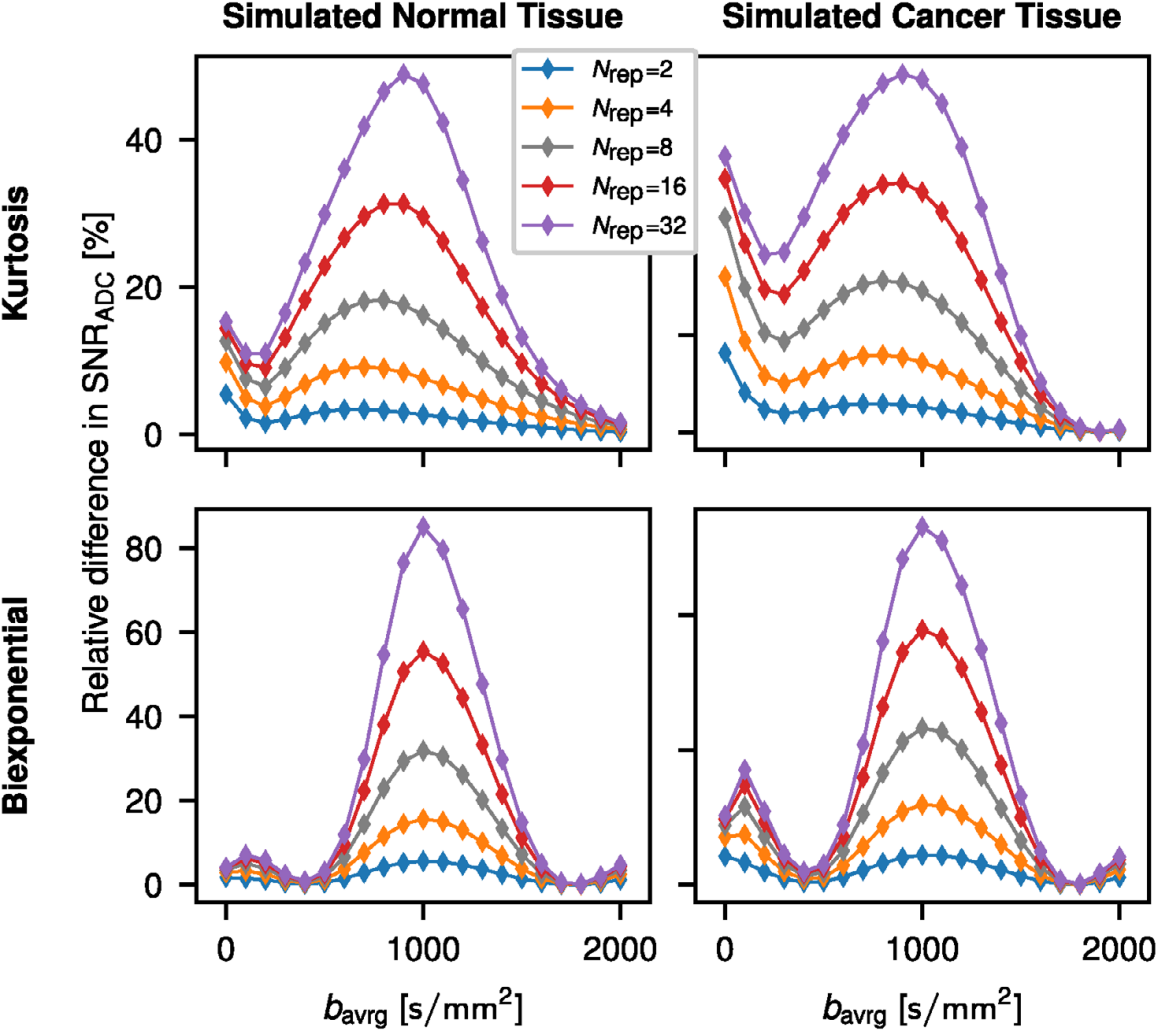
Improvement of ${\text{SNR}}_{\text{ADC}}$ for the retrospective ADC calculation as described in [2] for repeated measurements at a single b‐value. The relative ${\text{SNR}}_{\text{ADC}}$ difference (symbols) was determined for different levels of signal averaging ($N_{\text{rep}}$) applied individually at each of the 21 measured b‐values with respect to a single measurement ($N_{\text{rep}}=1$) at each b‐value. The retrospective ADC was calculated at b‐values of 100 and $1000\;s/{\text{mm}}^{2}$. Solid lines are visual guides.

The effect of signal averaging at a certain b‐value for the retrospective two‐b ADC calculation is shown in Figure 2. Best relative improvement is achieved in all cases for averaging around $b=1000\;s/{\text{mm}}^{2}$, which is the high b‐value in the two‐b ADC determination.

**FIGURE 3:**
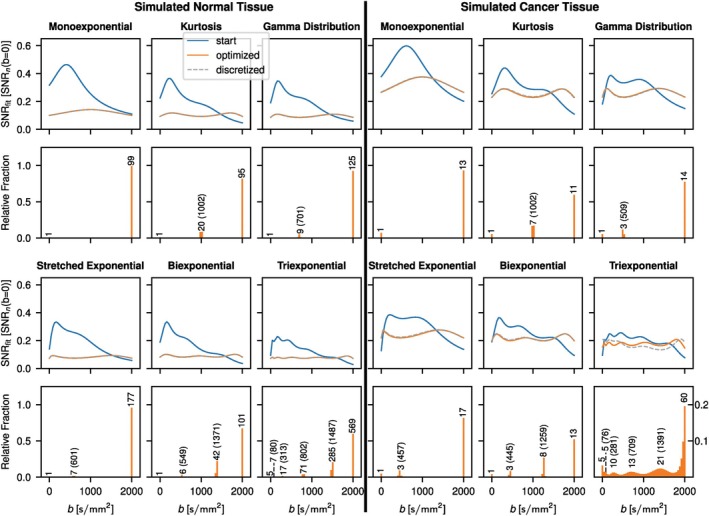
Optimized b‐value frequencies to approximate constant ${\text{SNR}}_{\text{fit}}$ over the whole b‐value measurement range. Results for normal and cancer example tissue are shown. ${\text{SNR}}_{\text{fit}}$ curves for uniformly distributed b‐values used as starting point for the optimization (start), optimized b‐value distributions (optimized) and discretized b‐value distributions (discretized) are plotted. In the histograms showing the optimized b‐value frequencies, the number of repeated measurements for each peak used in the discretized case is indicated. Optimized b‐values are indicated in parentheses, except for b=0 and $2000\;s/{\text{mm}}^{2}$, which are part of every optimized scheme. ${\text{SNR}}_{\text{fit}}$ is plotted in units of ${\text{SNR}}_{n}(b=0)$ defined as the resulting SNR if all measurements for a given case were performed at b=0. In the case of the discretized distribution this means that ${\text{SNR}}_{n}(b=0)=S_{0}/(\sqrt{n}\sigma_{g})$ with n being the sum over the repetitions at each b‐value as given in the corresponding histogram. This normalization allows for the direct comparison of all the ${\text{SNR}}_{\text{fit}}$ curves irrespective of the total amount of measurements n in the discretized case.

**FIGURE 4:**
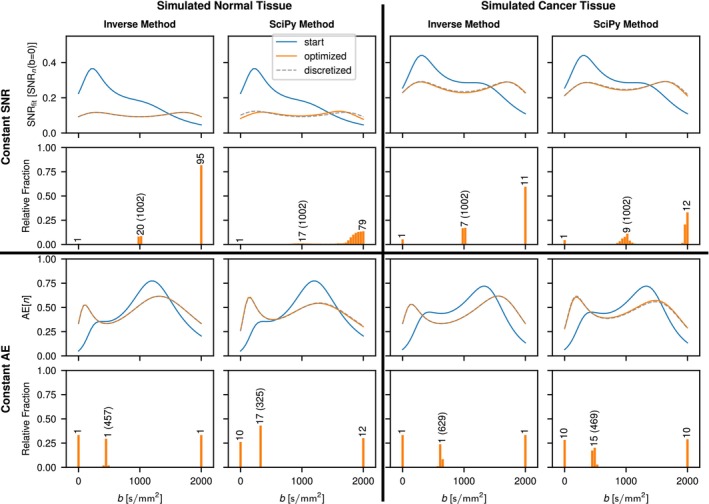
Comparison of Inverse and SciPy method for optimization of b‐value sampling for constant ${\text{SNR}}_{\text{fit}}$ and constant AE with a kurtosis model fit. As in Figure 3, the corresponding number of repeated measurements for the discretized case is given in the histograms for each peak. Optimized b‐values are indicated in parentheses, except for b=0 and $2000\;s/{\text{mm}}^{2}$, which are part of every optimized scheme. ${\text{SNR}}_{\text{fit}}$ is normalized by ${\text{SNR}}_{n}(b=0)$, the resulting SNR if all measurements for a given case were performed at b=0. AE is normalized by n, which in the discretized case corresponds to the sum over all repetitions at each b‐value as given in the corresponding histogram.

Results for the optimized b‐value schemes that aim to achieve constant ${\text{SNR}}_{\text{fit}}$ over the entire measured b‐value range are shown in Figure 3. With the exception of the triexponential model function applied in simulated cancer tissue, the b‐value distribution for the optimized scheme is characterized by discrete b‐values. Consequently, ${\text{SNR}}_{\text{fit}}$ curves for optimized and discretized schemes match. For the triexponential signal decay in cancer, the distribution is continuous, thus resulting in a poorer match of SNR(b) between the optimized scheme and the discretized scheme. The number of maxima in the optimized ${\text{SNR}}_{\text{fit}}$ curves is equal to the model parameter count excluding $S_{0}$. Furthermore, the position of the minima coincides with the optimized b‐values. Comparisons of the inverse and the SciPy method in the case of the kurtosis model are shown in Figure 4 for constant ${\text{SNR}}_{\text{fit}}$ and constant AE approximations. For both constant ${\text{SNR}}_{\text{fit}}$ and constant AE, there is good agreement between the two methods; however, the b‐value distributions that result with the SciPy method in the case of constant ${\text{SNR}}_{\text{fit}}$ appear less discretized.

**FIGURE 5:**
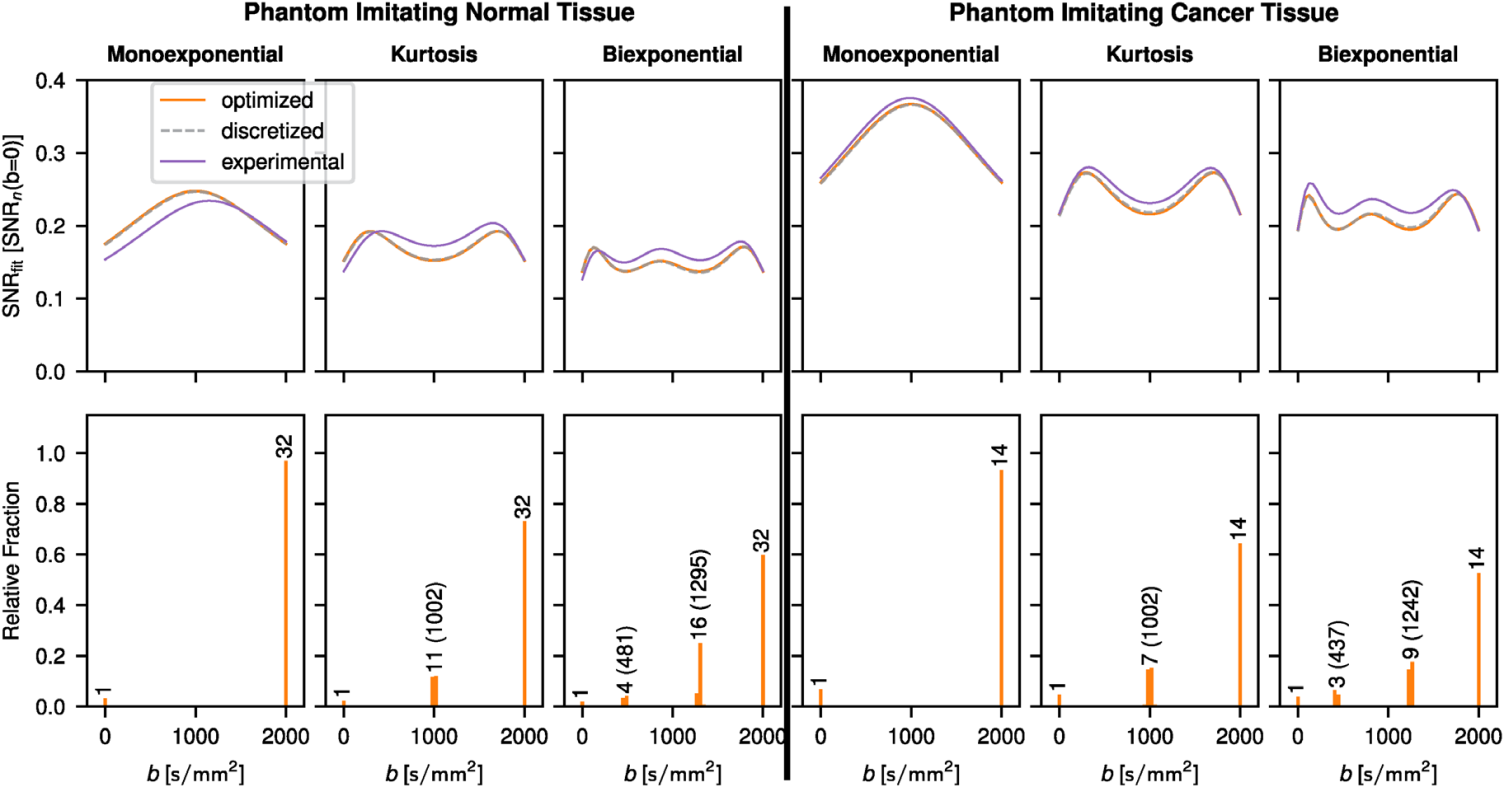
Comparison of theoretical and experimental results. The model parameters for the theoretical calculations were determined from the experimental data and differ slightly from the true model parameters used otherwise in this work as shown in Table S1. Deviations of the experimental results at b=0 for normal tissue can be explained by a higher experimental standard deviation at b=0 in comparison to the higher b‐values. The same normalization as in Figure 3 is applied, however, ${\text{SNR}}_{n}(b=0)$ is defined as $S(b=0)/\sqrt{n}\sigma (b=2000)$ in the experimental case due to this discrepancy (see also Figure S3). Again, for each peak the corresponding number of repeated measurements for the discretized case is given in the histograms. Optimized b‐values are indicated in parentheses, except for b=0 and $2000\;s/{\text{mm}}^{2}$, which are part of every optimized scheme.

Results for the phantom measurements are shown in Figure 5. Generally, there is a good agreement between theory and experiment. Deviations in the case of normal tissue at low b‐values are likely caused by a higher standard deviation of the repeat measurements of the ROI averaged signal in the corresponding phantom (see Supporting Information: Figure S3).

## 4 Discussion

Reducing noise in MR images by means of model fitting has been proposed recently [2, 8]. Instead of relying on repeated measurements at specific b‐values to obtain images of diagnostic value, measuring a larger number of b‐values without averaging followed by synthetic signal generation by means of model fitting, is a viable option. As shown in this work, the averaging effect, which is a measure of the noise reduction at a specific b‐value, depends in a non‐trivial manner on the signal model and its underlying parameters. Generally, the mean averaging effect over the studied b‐range is inversely proportional to the number of model parameters, with a global maximum found at a higher or the highest b‐value. However, models with a small number of model parameters, such as the monoexponential model, might not adequately describe the signal decay. Adding repeated measurements at a single b‐value increases the AE locally and can be a means of improving ${\text{SNR}}_{\text{fit}}$ for a certain b‐value of interest. Also, the uncertainty of the retrospective ADC analysis benefits from averaging at specific b‐values and can improve the quality of ADC maps.

Furthermore, we have shown that acquisition schemes can be constructed in a way that an almost constant ${\text{SNR}}_{\text{fit}}$ over a range of b‐values can be achieved for a certain tissue type. Interestingly, the number of suggested measuring points corresponds to the number of model parameters. As expected, the number of repetitions increases with the level of diffusion weighting. Compared to the uniform sampling strategy, the optimized constant SNR acquisition schemes come at the cost of a lower ${\text{SNR}}_{\text{fit}}$. This is particularly evident for the monoexponential function. For the other functions, the SNR gain is only consistent for the upper end of the measured b‐value range. The SciPy method gives similar results to the inverse method. However, the inverse method leads to distributions with narrower peaks and is considerably faster in computation. Constant fitting SNR could be of potential interest in cases where image quality measured in terms of SNR is preferred to be equivalent over a range of b‐values. Or one can aim for sampling schemes where SNR does not fall under a certain threshold. The analytic tools provide means to assess the costs and benefits of different sampling schemes, without having to resort to time‐consuming experiments. Even though not shown here, we found that the cost function (Equation (11)) can be adapted to optimize for other, such as a linear or quadratic, dependence of ${\text{SNR}}_{\text{fit}}$ with respect to b‐value.

Theoretical results have been verified by means of bootstrapping with experimental results from a phantom exhibiting non‐monoexponential signal decay with model parameters close to those found for real prostate tissue [5, 8]. Moreover, it shall be pointed out that the AE and the optimized schemes are independent of SNR, therefore the analysis is valid for any given SNR regime as long as signal distribution can be regarded Gaussian. For very low SNR (SNR<5), Rician signal distribution [9] needs to be considered for MR magnitude images requiring a dedicated analysis. Another limitation concerns the lack of diffusion anisotropy, even though it is regarded small for peripheral zone prostate tissue, where most tumors occur [10, 11]. Also an IVIM component is not present in the prostate mimicking phantom. Moreover, inclusion of clinical data would be helpful for translational goals. Finally, the effect of additional denoising, apart from model fitting, with MP‐PCA [12] or AI‐based algorithms could be contemplated, even though this might be difficult to include in the theoretical framework presented here.

## 5 Conclusions

With the help of analytical models, we have shown that a set of mathematical expressions can be used for the prediction of synthetic image SNR generated by means of model fitting and for sequence optimization in various aspects. We believe this work is of interest for developing novel acquisition schemes.

## Funding

This work was supported by the Cancerfonden, Vetenskapsrådet and the Swedish Governmental Funding of Clinical Research (ALF) (ALFGBG 932648), the Swedish Research Council, the Swedish Cancer Society, and NIH (R01CA241817, P41EB028741), Barncancerfonden.

## Disclosure

The authors have nothing to report.

## Conflicts of Interest

The authors declare no conflicts of interest.

## Supporting information




**Data S1. Figure S1.** Equivalent to Figure 1a but with more parameters and all studied model functions.
**Figure S2.** Approximation of model parameters for kurtosis, gamma distribution, and stretched exponential function from the biexponential normal and tumor tissue model.
**Figure S3.** Colored solid lines are ROI‐averaged signals for phantoms CSB05 (top) and CSB10 (bottom) as a function of time, with b‐values color‐coded. For each b‐value, the mean signal intensity has been subtracted. Solid black lines are quadratic fits of the respective signals. Dashed lines are drift‐corrected signals with the b‐value (in $s/{\text{mm}}^{2}$), and the signal standard deviation is indicated. Non‐drift corrected standard deviations are given in parentheses. Mean pixel‐wise SNR in ROIs at the highest b‐value was about 63 for CSB05 and 81 for CSB10. Excluded signals appear less saturated in color. Signals for b‐values of 10 and $100\;s/{\text{mm}}^{2}$ were not used in further analysis.
**Figure S4.** 2D image of the CSB05 (left) and CSB10 (right) phantoms in the water bath at b=0 along with the ROIs colored in red and green, respectively. Two additional phantom bottles in the waterbath (top and bottom) were not considered in this work.

## Data Availability

The data that support the findings of this study are available from the corresponding author upon reasonable request.

## Appendix A

### Data Errors

Data errors $\sigma_{y}$ for a model function y(x;a) with M free parameters given by vector $a=[a_{1},a_{2},\ldots ,a_{M}]$ can be approximated with an analytical expression as given by Richter [3] in the case of non‐linear least square estimation:

(A1)
$$\sigma_{y}^{2}(x;a)=\overset{M}{\underset{j=1}{\sum }}\overset{M}{\underset{k=1}{\sum }}C_{jk}(a)d_{j}(x;a)d_{k}(x;a).$$

The matrix C is the inverse H, that is,

(A2)
$$C=H^{-1},$$

where

(A3)
$${\left(H\right)}_{jk}=\overset{N}{\underset{i=1}{\sum }}\frac{1}{\sigma_{i}^{2}}d_{j}(x_{i})d_{k}(x_{i}),$$

and

(A4)
$$d_{l}(x)=\frac{\partial y}{\partial a_{l}}(x),$$

$x_{i}$ with i=1,…,N are the points where the measurements are taken, and $\sigma_{i}$ is the Gaussian noise standard deviation for the respective measurement point. Note that the matrix defined in Equation (A2) is identical to the covariance matrix used for the calculation of the Cramér–Rao lower bound [13] in the case of Gaussian noise.

Expressions for biexponential, kurtosis, and gamma distribution function are found in [2]. In the case of the stretched exponential function (Equation (6)) the derivatives with respect to the free parameters Equation (A4) are given by:

(A5)
$$\begin{array}{ll} d_{1}(x) & =\frac{\partial y}{\partial a_{1}}(x)=e^{-{\left(xa_{2}\right)}^{a_{3}}},\; \end{array}$$

(A6)
$$\begin{array}{ll} d_{2}(x) & =\frac{\partial y}{\partial a_{2}}(x)=-a_{1}a_{3}x{\left(xa_{2}\right)}^{a_{3}-1}e^{-{\left(xa_{2}\right)}^{a_{3}}}, \end{array}$$

(A7)
$$\begin{array}{ll} d_{3}(x) & =\frac{\partial y}{\partial a_{3}}(x)=-a_{1}{\left(xa_{2}\right)}^{a_{3}}\log(xa_{2})e^{-{\left(xa_{2}\right)}^{a_{3}}}, \end{array}$$

using the following definition with respect to Equation (6): $a_{1}=S_{0}$, $a_{2}=\text{DDC}$ and $a_{3}=\alpha$. In the case of the triexponential function Equation (3):

(A8)
$$\begin{array}{ll} d_{1}(x) & =\frac{\partial y}{\partial a_{1}}(x)=a_{5}e^{-xa_{6}}+(1-a_{5})\left(a_{2}e^{-xa_{3}}+(1-a_{2})e^{-xa_{4}}\right) \end{array}$$

(A9)
$$\begin{array}{ll} d_{2}(x) & =\frac{\partial y}{\partial a_{2}}(x)=a_{1}(1-a_{5})\left(e^{-xa_{3}}-e^{-xa_{4}}\right)\; \end{array}$$

(A10)
$$\begin{array}{ll} d_{3}(x) & =\frac{\partial y}{\partial a_{3}}(x)=-a_{1}a_{2}(1-a_{5})xe^{-xa_{3}}\; \end{array}$$

(A11)
$$\begin{array}{ll} d_{4}(x) & =\frac{\partial y}{\partial a_{4}}(x)=-a_{1}(1-a_{2})(1-a_{5})xe^{-xa_{4}}\; \end{array}$$

(A12)
$$\begin{array}{ll} d_{5}(x) & =\frac{\partial y}{\partial a_{5}}(x)=a_{1}\left(e^{-xa_{6}}-a_{2}e^{-xa_{3}}-(1-a_{2})e^{-xa_{4}}\right) \end{array}$$

(A13)
$$\begin{array}{ll} d_{6}(x) & =\frac{\partial y}{\partial a_{6}}(x)=-a_{1}a_{5}xe^{-xa_{6}},\; \end{array}$$

with $a_{1}=S_{0}$, $a_{2}=f$, $a_{3}=D_{2}$, $a_{4}=D_{3}$, $a_{5}=f_{p}$ and $a_{6}=D+D^{*}$. In the case of the gamma distribution Equation (5) the terms read:

(A14)
$$\begin{array}{ll} d_{1}(x) & ={\left(1+a_{3}x\right)}^{-a_{2}}\; \end{array}$$

(A15)
$$\begin{array}{ll} d_{2}(x) & =-a_{1}\log(1+a_{3}x){\left(1+a_{3}x\right)}^{-a_{2}} \end{array}$$

(A16)
$$\begin{array}{ll} d_{3}(x) & =-a_{1}a_{2}x{\left(1+a_{3}x\right)}^{-a_{2}-1},\; \end{array}$$

with $a_{1}=S_{0}$, $a_{2}=k$ and $a_{3}=\vartheta$.
