# Supplementary material for: Optimization of Diffusion MRI With Consideration of the Signal Decay in Biological Tissues
Source: Magn Reson Med. 2026 Mar 19;96(1):460–8. doi: 10.1002/mrm.70346 (PMC13112234; doi:10.1002/mrm.70346)
Supplement: Supplementary file 1 — Data S1. Figure S1. Equivalent to Figure 1a but with more parameters and all studied model functions. Figure S2. Approximation of model parameters for kurtosis, gamma distribution, and stretched exponential function from the biexponential normal and tumor tissue model. Figure S3. Colored solid lines are ROI‐averaged signals for phantoms CSB05 (top) and CSB10 (bottom) as a function of time, with b‐values color‐coded. For each b‐value, the mean signal intensity has been subtracted. Solid black lines are quadratic fits of the respective signals. Dashed lines are drift‐corrected signals with the b‐value (in s/mm2), and the signal standard deviation is indicated. Non‐drift corrected standard deviations are given in parentheses. Mean pixel‐wise SNR in ROIs at the highest b‐value was about 63 for CSB05 and 81 for CSB10. Excluded signals appear less saturated in color. Signals for b‐values of 10 and 100s/mm2 were not used in further analysis. Figure S4. 2D image of the CSB05 (left) and CSB10 (right) phantoms in the water bath at b=0 along with the ROIs colored in red and green, respectively. Two additional phantom bottles in the waterbath (top and bottom) were not considered in this work. [file MRM-96-460-s001.pdf]

Received ; | Revised ; | Accepted
DOI: xxx/xxxx

SUPPORTIVE INFORMATION

Optimization of diffusion MRI with consideration of the signal decay in biological tissues

Stefan Kuczera[1] | Stephan E. Maier[1,2]

[1]Institute of Clinical Sciences, Sahlgrenska Academy,, University, Gothenburg, , Sweden
[2]Department of Radiology, Brigham Women's Hospital, Harvard Medical School, MA, Boston

Correspondence
Stefan Kuczera. Email: stefan.kuczera@gu.se

1 | SAMPLE CONTAINER

Samples were contained in plastic bottles of 7 cm height and 3 cm diameter. The plastic bottles were placed in a plastic container (L, W, H: 16 cm, 16 cm, 9 cm) filled with tap water and approximately 15 g of dissolved table salt. Pressure-sensitive adhesive between the top of the bottles and the container lid was used to prevent sample motion during the measurement.

□

TABLE S1 Models and model parameters associated with normal and cancerous tissue

Function	Parameter	NT Theory	CT Theory	NT Experimental	CT Experimental
monoexponential	D_m	1.15	0.645	0.863	0.657
biexponential	D_1	2.2	2.2	2.26	2.3
	D_2	0.4	0.2	0.3	0.21
	f	0.8	0.6	0.69	0.6
kurtosis	$\mathrm{ADC_K}$	1.866	1.296	1.5	1.24
	K	0.627	1.231	0.85	1.14
gamma distribution	ϑ	2.604	0.838	-	-
	k	0.763	1.963	-	-
stretched exponential	DDC	1.572	0.835	-	-
	β	0.830	0.678	-	-
IVIM	D_1	2.2	2.2	-	-
	D_2	0.4	0.2	-	-
	f	0.8	0.6	-	-
	$D + D^*$	12	12	-	-
	f_p	0.06	0.06	-	-

Diffusion coefficients and ϑ are in $\mu m^2/ms$

NT: Normal tissue; CT: cancerous tissue

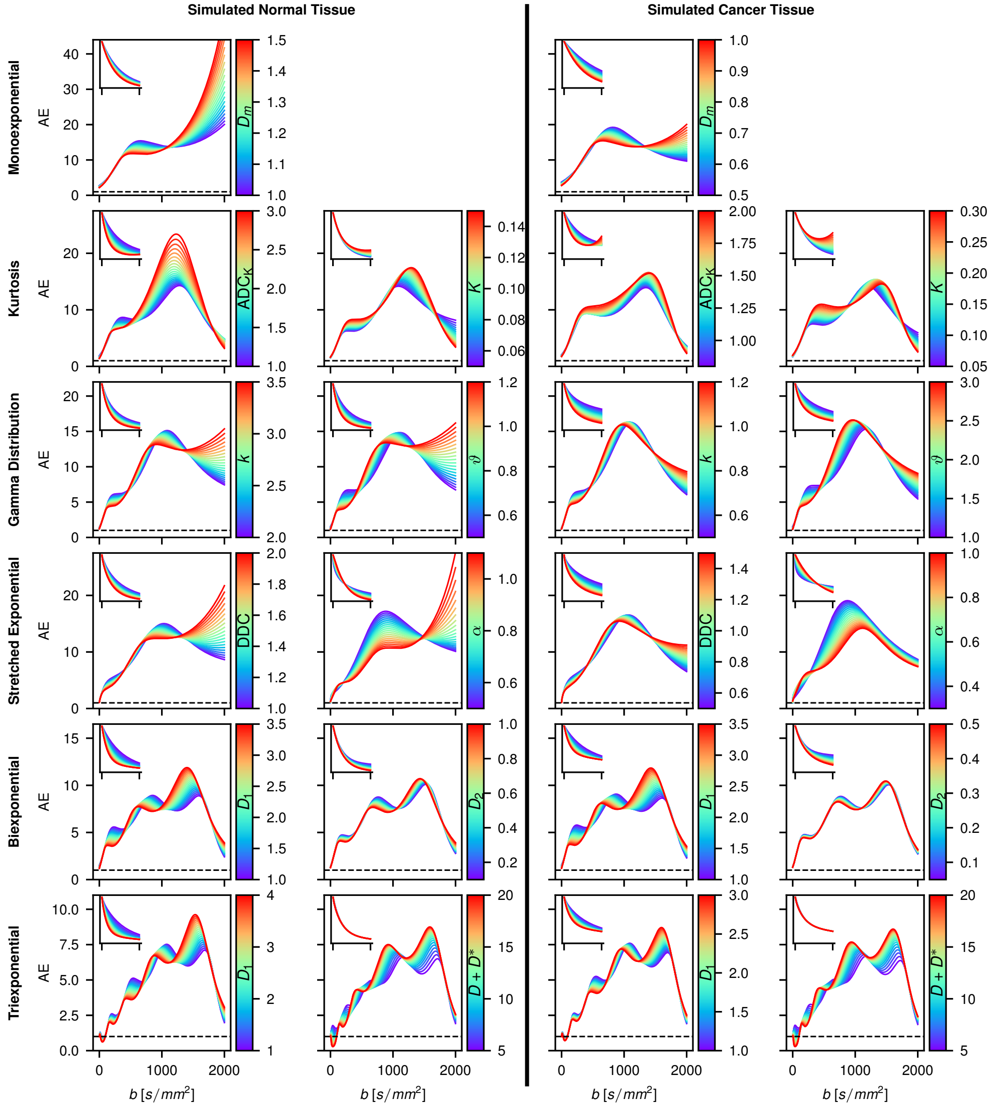

FIGURE S1 Equivalent to Figure 1 a but with more parameters and all studied model functions.

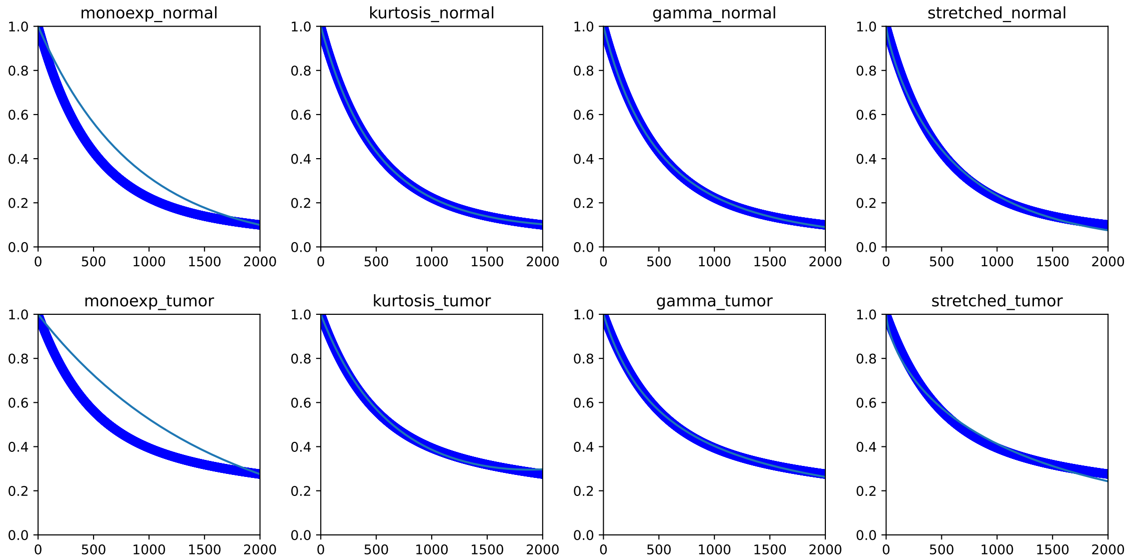

FIGURE S2 Approximation of model parameters for kurtosis, gamma distribution and stretched exponential function from the biexponential normal and tumor tissue model.

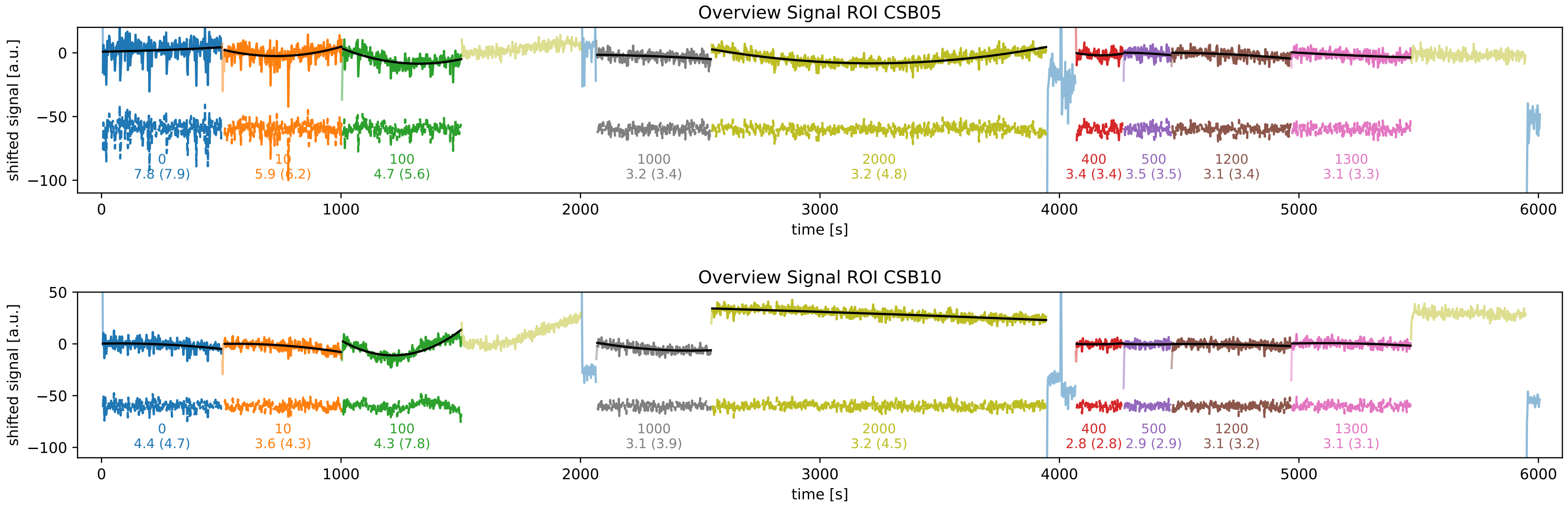

FIGURE S3 Colored solid lines are ROI-averaged signals for phantoms CSB05 (top) and CSB10 (bottom) as a function of time with b-values color-coded. For each b-value the mean signal intensity has been subtracted. Solid black lines are quadratic fits of the respective signals. Dashed lines are drift-corrected signals with the b-value (in s/mm^2) and the signal standard deviation indicated. Non-drift corrected standard deviations are given in parenthesis. Mean pixel-wise SNR in ROIs at highest b-value was about 63 for CSB05 and 81 for CSB10. Excluded signals appear less saturated in color. Signals for b-values of 10 and 100 s/mm^2 were not used in further analysis.

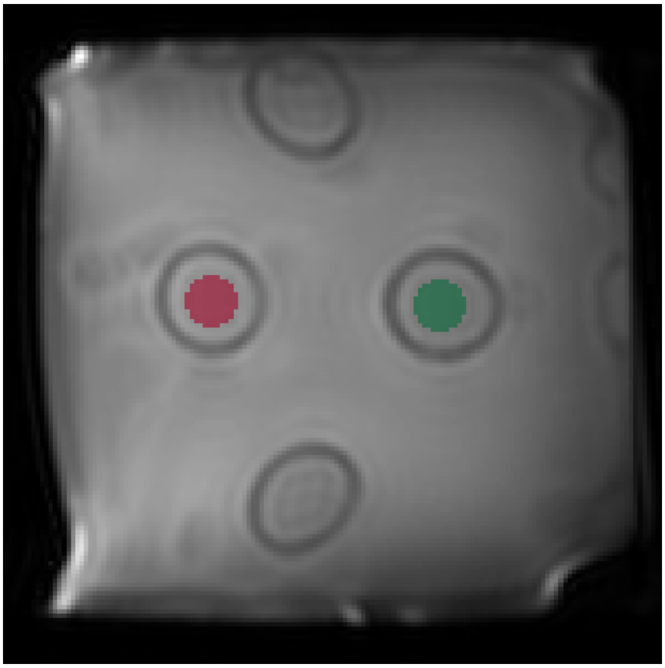

FIGURE S4 2D image of the CSB05 (left) and CSB10 (right) phantoms in the water bath at $b = 0$ along with the ROIs colored in red and green, respectively. Two additional phantom bottles in the waterbath (top and bottom) were not considered in this work.
